# An open-source, 3D printed inkjet DNA synthesizer

**DOI:** 10.1038/s41598-024-53944-x

**Published:** 2024-02-14

**Authors:** Junhyeong Kim, Haeun Kim, Duhee Bang

**Affiliations:** Department of Chemistry, Yonsei University, Seoul, Korea

**Keywords:** Biological techniques, Chemical biology

## Abstract

Synthetic oligonucleotides have become a fundamental tool in a wide range of biological fields, including synthetic biology, biosensing, and DNA storage. Reliable access to equipment for synthesizing high-density oligonucleotides in the laboratory ensures research security and the freedom of research expansion. In this study, we introduced the Open-Source Inkjet DNA Synthesizer (OpenIDS), an open-source inkjet-based microarray synthesizer that offers ease of construction, rapid deployment, and flexible scalability. Utilizing 3D printing, Arduino, and Raspberry Pi, this newly designed synthesizer achieved robust stability with an industrial inkjet printhead. OpenIDS maintains low production costs and is therefore suitable for self-fabrication and optimization in academic laboratories. Moreover, even non-experts can create and control the synthesizer with a high degree of freedom for structural modifications. Users can easily add printheads or alter the design of the microarray substrate according to their research needs. To validate its performance, we synthesized oligonucleotides on 144 spots on a 15 × 25-mm silicon wafer filled with controlled pore glass. The synthesized oligonucleotides were analyzed using urea polyacrylamide gel electrophoresis.

## Introduction

In recent decades, synthetic oligonucleotides(oligos) have played an important role in the field of biology^1,2^. High-density oligo arrays have shown significant potential in various applications, such as libraries, genetic building blocks^3,4^, on-chip analysis using microarrays^5–7^, and DNA data storage^8–10^. Various techniques based on photolithography^7,10,11^, inkjet printing^12–14^, and electrochemical methods^15^ have been developed for synthesizing high-density oligo arrays and have significantly reduced the synthesis cost per oligo.

Among these synthesis techniques, inkjet-based oligo synthesis using multichannel inkjet printheads allows for flexible sequence and array modifications by selectively dispensing DNA monomers. It offers the additional advantage of non-contact deposition, which eliminates contamination. Moreover, it enables high-yield synthesis based on standard phosphoramidite chemistry. However, despite these advantages, accessing inkjet synthesis equipment has been challenging for basic academic research laboratories: the commercially available oligo arrays are expensive and have limited customizability, thus restricting the possibilities of creative research.

Recently, the development of low-cost, open-source microcontrollers, such as Arduino and Raspberry Pi, along with 3D printing technology, has significantly lowered the barriers to the self-construction of experimental equipment in research labs^16,17^. Scientists have utilized these technologies to develop affordable and customizable devices, such as arthropod venom extractors^18^, microfluidic flow cytometers^19^, solid-phase peptide synthesizers^20^, RNA/DNA extractors^21^, and liquid-handling workstations^22^. However, there is insufficient research on the self-construction of microarray synthesizers, especially those based on inkjet technology. Therefore, we introduce the Open-Source Inkjet DNA Synthesizer , a low-cost, flexible, and open-source inkjet DNA synthesis device. It provides guidelines for performing cost-effective oligo synthesis using open-source microcontrollers. We developed an assembly-ready synthesizer using 3D printed structures and commonly available components. OpenIDS utilizes inkjet technology to offer user-friendly operation, affordability, and high precision and fidelity. It also includes a graphical user interface (GUI) that enables researchers to easily control, modify, and monitor the synthesis process. We aim to provide a practical solution for academic laboratories and researchers lacking expertise in electrical and mechanical engineering who wish to self-assemble a microarray synthesizer for specific modifications to the sequence and substrate material of microarrays or to adjust the size of the synthesizer according to their needs.

## Results

### Development of the DNA microarray synthesizer

OpenIDS was designed with aluminum profile frameworks, allowing for easy assembly and disassembly of the six-sided acrylic covers and convenient modification and sealing for synthesis purposes. These are easy to modify following the user’s needs and to seal with nuts and bolts. The synthesizer described here was built with dimensions of 830 × 480 × 480 mm, but it can be easily adjusted to the desired size. OpenIDS consists of five main modules: (1) an inkjet printer head for dispensing the activator and A, T, G, C phosphoramidite (referred to as ink) according to the desired nucleotide sequence, (2) an ink supply system to deliver ink to the printer head, (3) syringe pumps to deliver reagents for the wash, oxidation, and detritylation steps, (4) a linear actuator for positioning the synthesis substrate at the proper reagent dispensing locations, and (5) a camera for monitoring the printing process.

**Figure 1:**
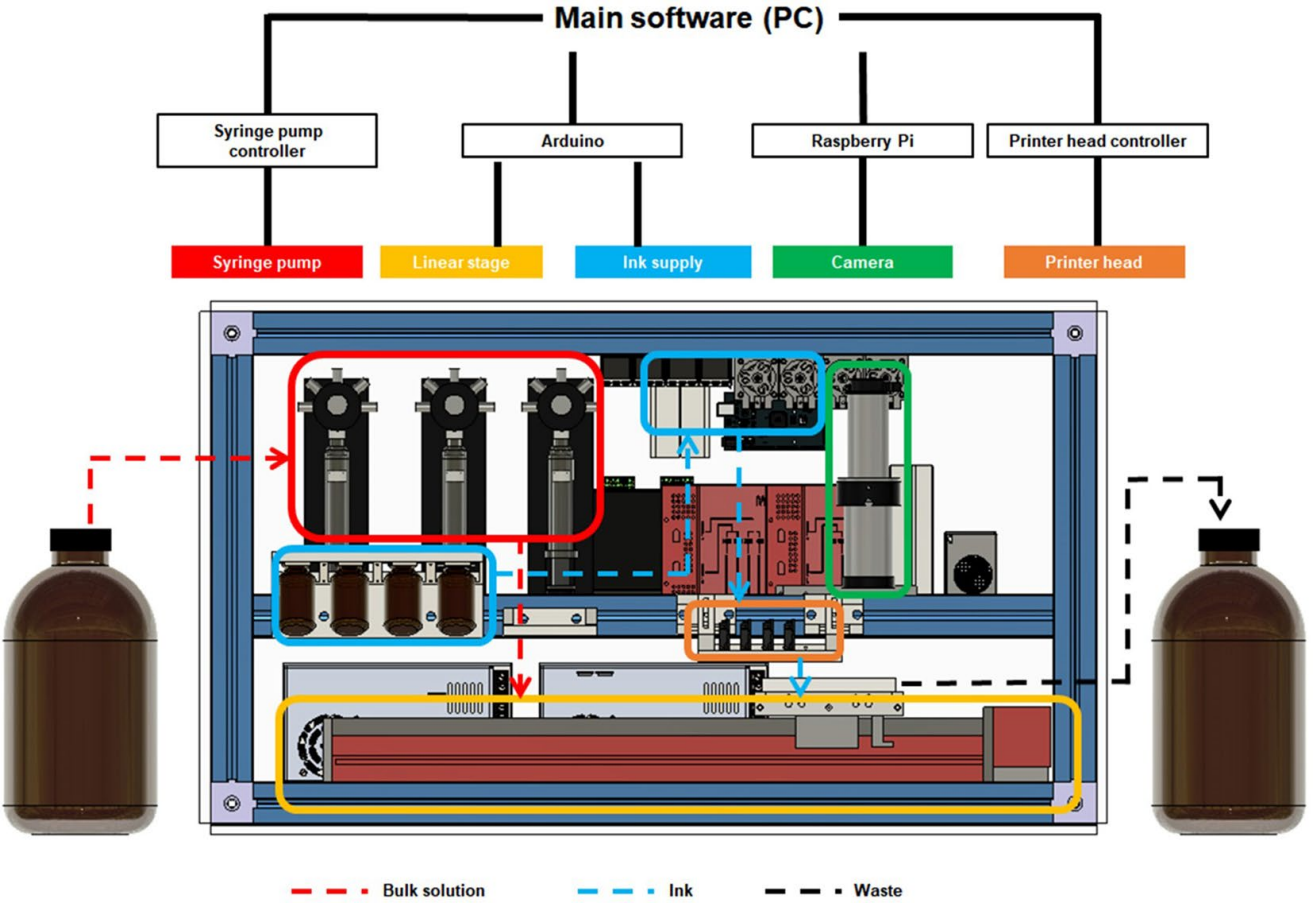
The design of OpenIDS. It consists of five major hardware modules. Each module is controlled by four controllers, which are integrated into the main software of the PC. The dotted arrows show the fluid flow of each reagent during oligo synthesis. The linear slide transports the synthetic substrate to dispense each reagent.

We configured appropriate controllers for each of the five modules to establish an organic connection and integrated them into a single main control program (Fig. 1). Each syringe pump module was equipped with an integrated controller. Arduino controls the linear actuator and ink supply system, and Raspberry Pi controls the camera. The main software program, which was written in Python and C, integrates all the modules to ensure their organic collaboration. The flow of the reagents during oligo synthesis is shown by the dotted arrows in Fig. 1. The interior of the synthesizer was sealed with acrylic covers to prevent oxygen and moisture infiltration, which could interfere with the synthesis process. We filled the synthesizer with nitrogen(N_2_) or argon(Ar) gas through the inlet on the left side of the synthesizer, and it exited through the outlet on the opposite side. The gas helps to decrease the humidity level to 0% and maintain that level during the synthesis process. During the coupling step, the synthesis substrate passes under the printhead, where the appropriate nucleotides are printed. In the wash, oxidation, and detritylation steps, the substrate moves to the corresponding positions for dispensing each reagent. For precise reagent delivery, the bulk solution was dispensed using a syringe pump with teflon tubes. The N_2_ or Ar gas, controlled by a solenoid valve, was used to dry the synthesis substrate after the wash step. Solenoid valves are commonly used for reagent delivery in synthesizers due to their small size, affordability, and ease of control. However, using valves to dispense reagents requires a supply of positive pressure, and excessive pressure can lead to valve malfunctions and leakage of hazardous reagents. We aim to provide a guide for easy self-construction in individual laboratories to minimize the risk of such construction problems.

**Figure 2:**
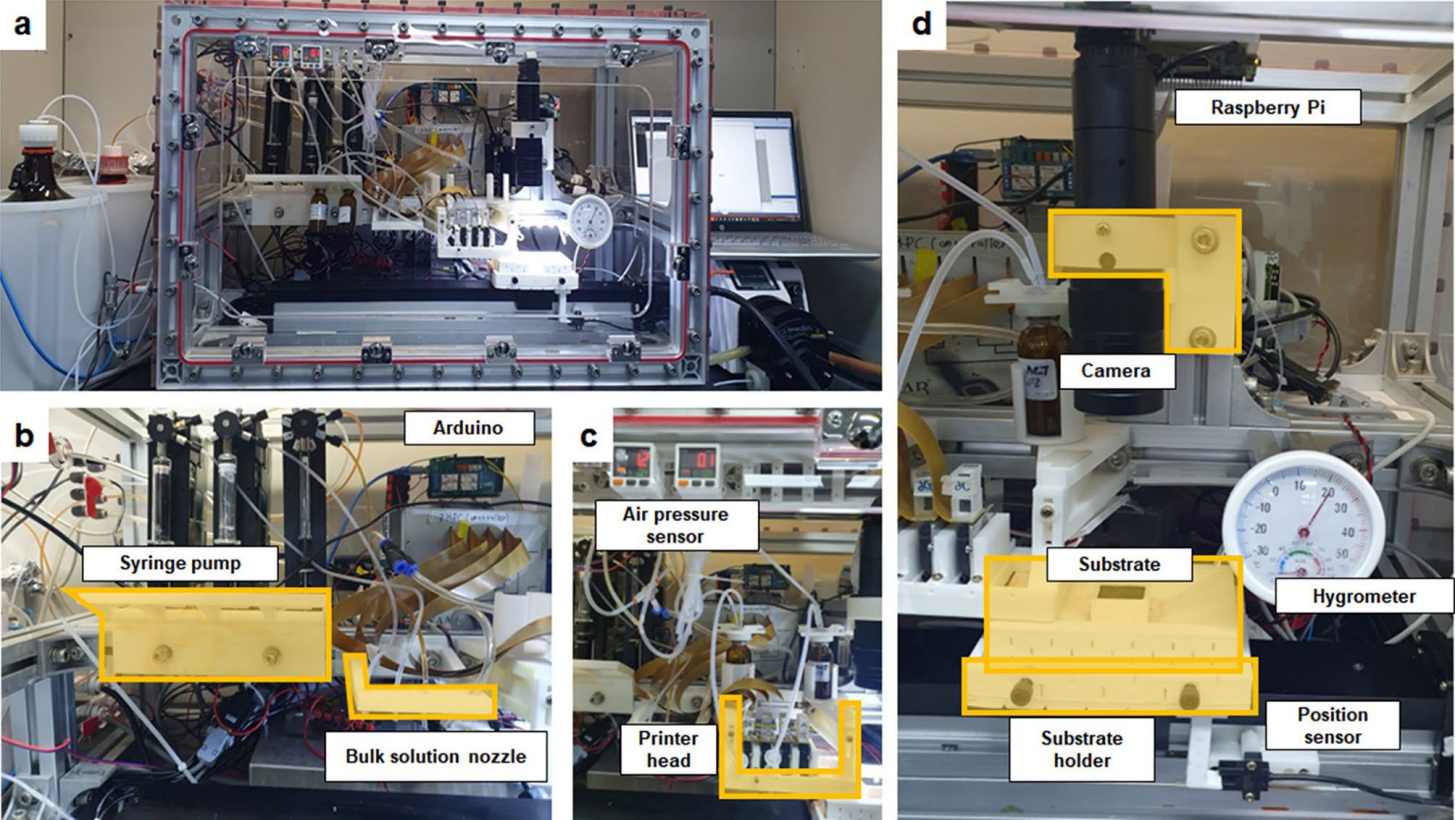
The OpenIDS platform. The elements highlighted by the yellow boxes were 3D printed. (**a**) Overview. The frame of the synthesizer is made of aluminum profiles and acrylic. The bulk solution is stored to the left of the synthesizer, and to the right are a laptop computer for controlling the synthesizer and a pump for waste liquid removal. (**b**) The syringe pump draws bulk solution from outside the synthesizer and dispenses it onto the synthesizer substrate. (**c**) The ink is delivered to the printhead via positive pressure to the ink bottle using a linked pump. The pressure sensor maintains the appropriate pressure of the ink bottle for optimal printing. (**d**) The substrate is detached and transferred horizontally on the substrate holder. After each coupling step is completed, the substrate holder moves below the camera so the user can check the print quality. The substrate holder was 3D printed using polypropylene for chemical resistance.

The actual appearance of the built OpenIDS is shown in Fig. 2. The elements highlighted with yellow boxes in the figure were 3D printed. The substrate holder, which directly contacts the reagents, was printed with polypropylene (PP) for chemical resistance. Other structures were made with polylactic acid(PLA), the most commonly used material in 3D printing. The controllers, including the pumps, relays, Arduino, and Raspberry Pi, are located on the upper part of the synthesizer (Fig. 2b–d). In the lower part, the reagents dispense and synthesize oligonucleotides. The substrate is mounted on the substrate holder, which is fixed on the linear actuator and moves to the appropriate positions for dispensing bulk solution along the x-axis. The air pressure sensor regulates the ink supply pressure for successful jetting. The amidite bottle, air pressure sensor, peristaltic pump, and printhead are connected by silicone tubes. The peristaltic pumps supply positive pressure to the amidite bottle to push the reagents toward the printhead. The 3D designs of all components, including the structures created through 3D printing, are available to download through GitHub^23^. The total cost for the assembly of the OpenIDS with five printheads is around $19,900. This is more budget-friendly compared to the production cost of POSaM, a previously researched open-source inkjet DNA synthesizer, which was $34,000^13^. Additionally, it competes favorably with the cost of acquiring a second-hand commercial DNA synthesizer based on a well plate ($15–30K). See Supplementary Table 1 for an aspect-by-aspect comparison. The components of OpenIDS along with their respective prices are listed in Supplementary Table 2.

### Inkjet printing of phosphoramidite

**Figure 3:**
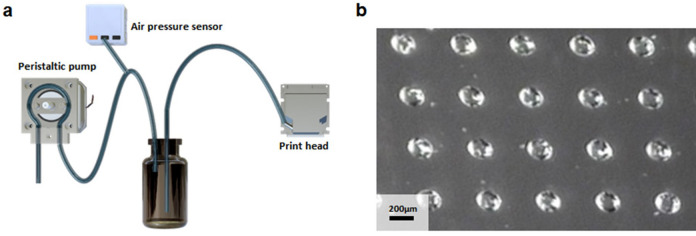
Inkjet printing of phosphoramidite. (**a**) The peristaltic pump rotates clockwise, pushing air into the ink bottle. The ink inside the bottle is flushed to the printhead. Next, the pump rotates counterclockwise, expelling air and adjusting the pressure inside the bottle to an appropriate level. The air pressure sensor is connected to the peristaltic pump to measure the internal pressure of the bottle. (**b**) Photomicrograph of silanized glass; 0.25 M phosphoramidites and 0.32 M ETT in propylene carbonate were printed onto standard microscope slides coated with hydrophobic silane. The scale bar at the bottom left corresponds to 200 µm.

DNA array synthesis was performed by phosphoramidite chemistry, which eliminates the capping step. In this chemistry, acetonitrile (ACN) is commonly employed as a solvent for phosphoramidite and as a 5-(ethylthio)-1H-tetrazole (ETT) activator. However, due to its high volatility, acetonitrile is not suitable for inkjet-based synthesis: it cannot be maintained on the substrate during the coupling step and may even evaporate before reaching the synthesis substrate. Furthermore, it can damage the printhead. Propylene carbonate (PC) is a less-volatile alternative that has been shown to produce a coupling efficiency of 94%–98% for inkjet oligonucleotide synthesis^24^. To achieve successful printing, it is necessary to supply the ink at the appropriate pressure, which may vary depending on the printhead and ink used. We designed an ink supply system with flexible pressure control (Fig. 3a). Ink bottles connected to two tubes: one connected to a peristaltic pump and pressure sensor to regulate the internal pressure of the bottle, and the other connected to the printhead for the ink supply. The peristaltic pump provided a positive pressure of several kPa to fill the printhead with ink, allowing for approximately 3 ml of flushing. It then rotated in the opposite direction to maintain the desired pressure. To ensure successful printing of PC, our ink supply system requires a consistent pressure of 0.9 kPa. This value can vary depending on the relative positioning of the printhead and ink bottle as well as the size of the filter.

We successfully printed 0.25 M phosphoramidites and 0.32 M ETT in PC The diameter of the printed droplets was determined by measuring the size of 20 droplets captured through a 95× magnification microscope using ImageJ. The average diameter of the droplets was 227.5 µm, with a standard deviation of 8.9 µm, and a coefficient of variation of 0.039 (Fig. 3b). As PC is hygroscopic and DNA synthesis is highly susceptible to moisture, it is necessary to use fresh reagents for each synthesis. Therefore, we cleaned the printer head with pure, dry PC before and after each synthesis and then dried it with argon.

### Alignment of printheads

When integrating multiple printheads onto a single system, misalignment can occur due to spacing issues. If the printheads are not precisely aligned, it can lead to sequencing errors at the edges of features, as the activator and bases may not be injected at the same position. This is commonly known as the ‘edge effect’, and it becomes a more critical source of errors as features decrease in size.

**Figure 4:**
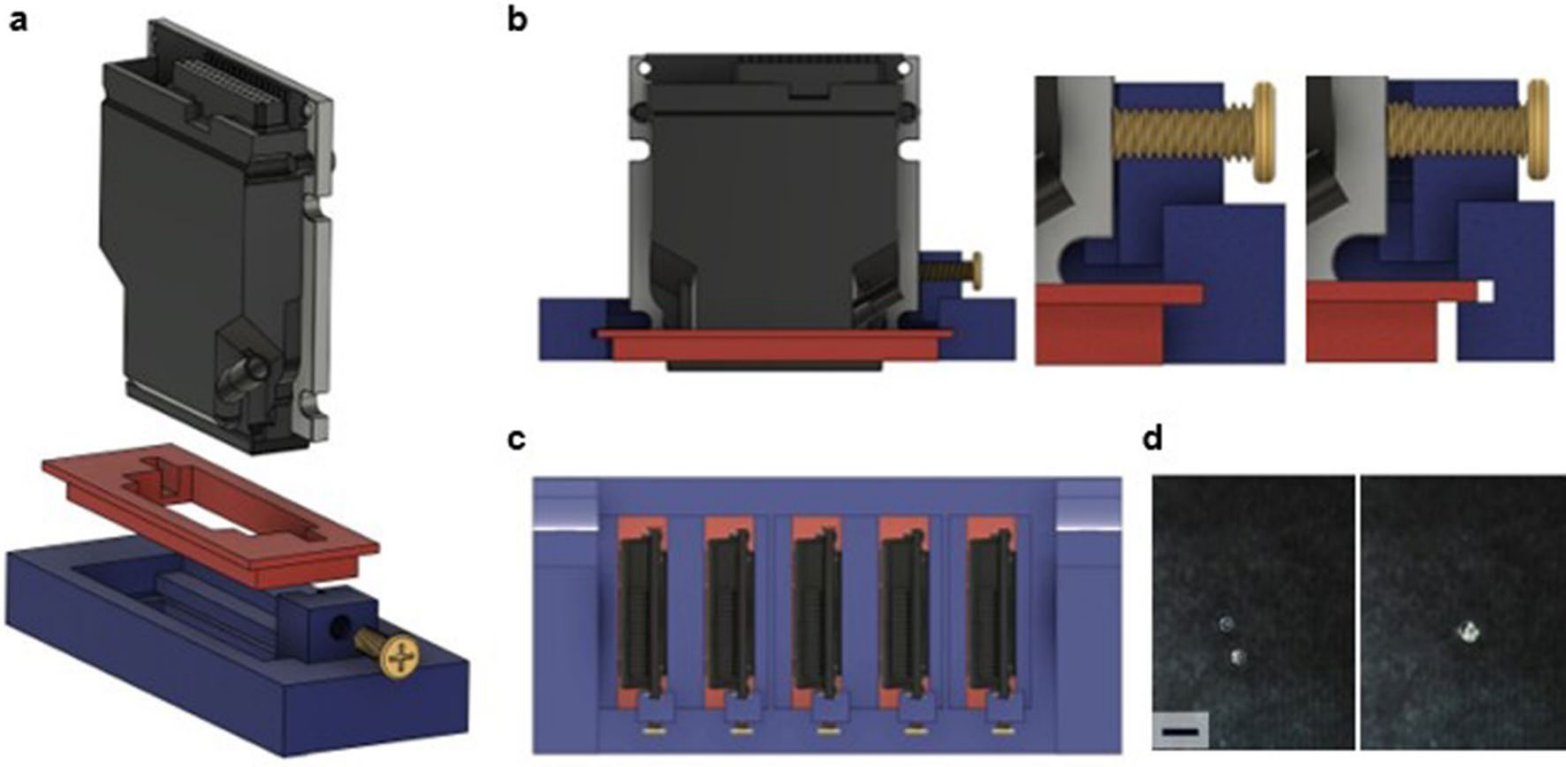
Alignment of the printhead. To achieve precise alignment of the printheads, printhead cluster is designed with a mechanism for fine-tuning printheads’ physical position using bolts. (**a**) The printhead is mounted on the main body through a sliding holder (red part) that allows forward and backward movement. The sliding holder is 3D-printed in PP, and its inherent flexibility ensures a secure fit between the printhead and the main body. (**b**) The main body features screw threads, and the printhead attached to it can be adjusted by pushing it using M3 screws. The pitch of the M3 screw is 500 µm, enabling precise adjustments at the micrometer level between the printhead and the sliding holder. Once adjustments are complete, the PP holder is naturally secured in place by the friction, holding it firmly against the main body. (**c**) A top-angle view of the printhead cluster. Starting with the leftmost printhead as the reference point, each printhead is sequentially aligned through print tests. (**d**) Droplets printed from different printheads at the same position exhibited positional errors before alignment, but after alignment, they were consolidated into a single droplet. The scale bar at the bottom left corresponds to 100 µm.

The printhead requires alignment in two main directions: along the nozzle line and perpendicular to it. As printing progresses, the substrate moves in the direction perpendicular to the nozzle line, and each printhead injects ink with a time delay synchronized to the board's movement speed. Therefore, simply adjusting this time delay allows for easy alignment in the direction perpendicular to the nozzle line. Alignment in the direction along the nozzle line requires a separate physical alignment. The minimum unit adjustable in this direction through software is the spacing between nozzles, which is 137.1 μm in our system. As this level of precision is unsatisfactory for the fabrication of microarrays, we have designed a structure for the physical alignment of the printheads (Fig. 4). To enable the movement of the printhead in the nozzle-line direction, the printhead is initially coupled to a sliding holder made of PP material before being integrated into the main body (Fig. 4a). The flexible PP material wedges into the crevice of the main body like rubber, securing it through friction. Slowly tightening the screw on the main body pushes the printhead, allowing for extremely fine adjustments along with the PP holder (Fig. 4b). Using M3 screws with a pitch of 500μm, we could adjust the position of the head in increments of several micrometers. Using the leftmost printhead as a reference, after several iterations of printing, observing, and adjusting, we successfully aligned the remaining printheads to print the same single point (Fig. 4c,d).

### User-friendly GUI-based synthesis software

**Figure 5:**
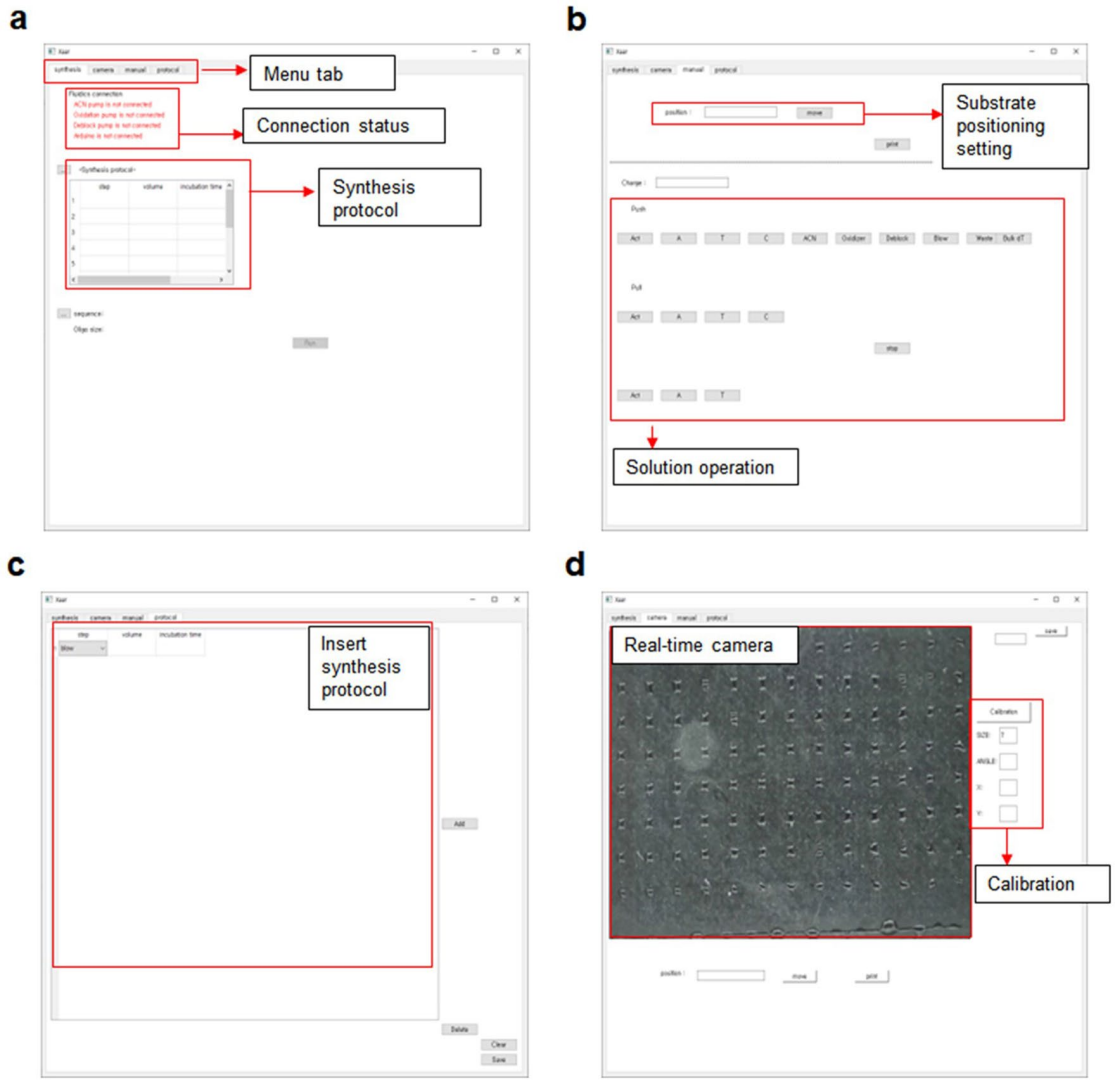
Synthesis software GUI. The main software was programmed using Python. It integrates C-based Arduino and a printhead controller as well as other controllers. Our software allows users to simply utilize the system by editing or programming new Python scripts. The software (**a**) displays the connection state of each module and load synthesis protocol, (**b**) gives users manual control of each solution and linear actuator, (**c**) allows users to write or edit synthesis protocols, and (**d**) provides a real-time monitoring and manual calibration system.

The main software of the synthesizer was developed using Python, a programming language known for its extensive libraries and easy, beginner-friendly code structure^25^. Python was adopted as the main software language because it allows for easy customization of the software for different laboratory environments and experiments. Although the Arduino and printhead operation codes were written in C for high performance, users can adjust the operation of all hardware components by simply modifying the top-level Python script. The software’s GUI is essential because it enables users with no understanding of the overall software structure to easily operate the equipment. It is common for researchers to face months of trial and error when trying to apply open-source equipment and analysis tools in practice due to unintuitive code. OpenIDS provides a complete GUI-based software that allows users to control each component, including its connection status, and define synthesis protocols. Additionally, users can generate, modify, and observe the synthesis process in real time through the camera (Fig. 5). The Python scripts for operating the synthesizer are available to download through GitHub^23^.

### Silicon wafer–based substrate

**Figure 6:**
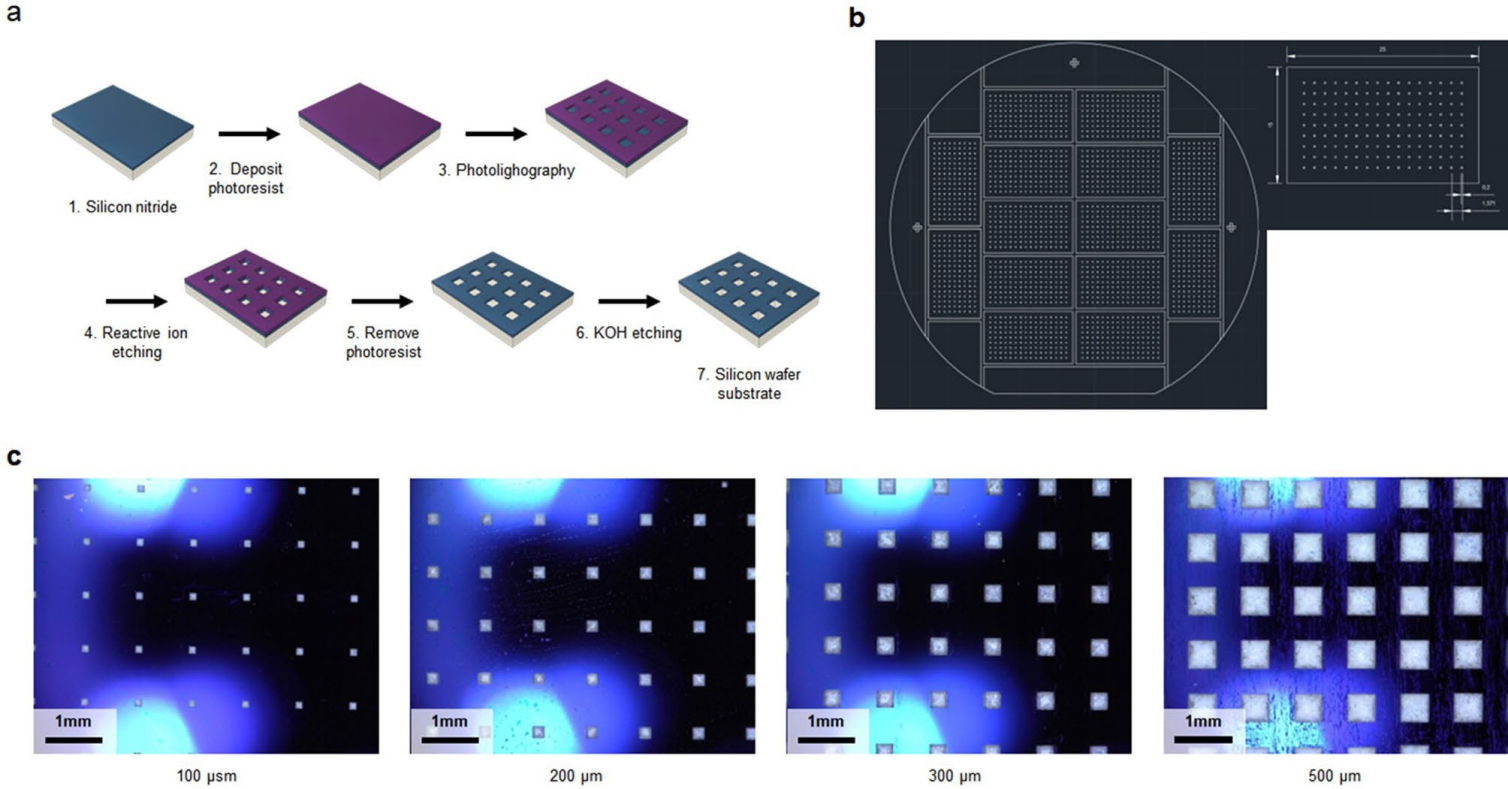
Silicon wafer–based substrate. The wafers were modified with photolithography. (**a**) The six steps for making the wafer-based substrate. The final step involves KOH etching to create uniform trapezoid microholes. The diagram shows a simple arrangement of 4 × 3 wells on a wafer; however, our process produced a 16 × 9 grid of wells. (**b**) The photomask contained the desired fine pattern, which was transferred onto the silicon wafer surface using photolithography. In this study, the photomask design included 14 pieces measuring 25 × 15 mm each. (**c**) Well sizes of 100, 200, 300, and 500 µm on a silicon wafer. The center-to-center distance of the features was 1371 µm. The scale bar at the bottom left corresponds to 1 mm. A silicon wafer is dark bluish because of the silicon nitride surface. The white square dots represent features filled with CPG.

The substrate can be any type of material that has a surface, chemical resistance, and easy to modify. In this study, a silicon wafer was chosen because of its chemical resistance and easy modification. The silicon wafer can be scalable using photolithography. By taking advantage of the inkjet printer’s rapid and precise dispensing onto a surface, we formed a DNA synthesis spot on the surface by using a silicon wafer (Fig. 6a).

The nitride layer was deposited on a 4-in silicon wafer surface using low-pressure chemical vapor deposition (LPCVD). A layer of photoresist was uniformly deposited on the nitride silicon wafer surface. Then, the wafer was exposed to light through a photomask. This structure on the photomask was transferred onto the photoresist on the wafer’s surface. The photoresist corresponding to the structure was removed, and then nitride was exposed. Next, we performed reactive ion etching (RIE) and selectively removed the resulting exposed nitride. The remaining photoresist was removed using acetone and water. The silicon was etched with a 30% potassium hydroxide (KOH) solution at a controlled angle, forming a trapezoidal well. Each well is called a feature that is a location for oligonucleotide synthesis. We obtained 15 × 25-mm 14 pieces from a 4-in wafer and the production cost for one piece was $7.50 (Fig. 6b, Fig. S1, Table. S4) The wafer pieces serve as an oligo synthesis substrate. It was cleaned with distilled water and ethanol and then dried. For oligo synthesis, the wells were filled with controlled pore glass (CPG), which has functional groups that can aid in DNA synthesis. Clearly separated features allow the reagent to be efficiently delivered to the CPG inside without contamination. The prepared substrates were stored in a desiccator at room temperature.

In this study, each wafer piece had a 16 × 9 pattern of 200-μm squares, though the size and number of features are scalable. Due to the good chemical resistance of the wafer, it can withstand exposure to harmful reagents. 100-, 200-, 300-, and 500-µm square features were estimated (Fig. 6c). As the feature size increased, the consumption of reagents increased. Not only does it increase the usage of reagents, it also increases the number of printings, resulting in the total synthesis time also elongated. Based on our measurements, we suggest CPG’s mean diameter is approximately 100 µm. Therefore, the 100 µm feature is too small to effectively fill in CPG, as a result, it produces waste. We determined that 200 µm was the optimal feature size for easy-making oligo synthesis substrate. Through our observation, the oligo synthesis was focused on 200 µm features-silicon wafer substrate (Fig. S2). The distance between features was determined to be 1371 µm, which is 10 times the nozzle-to-nozzle distance in the printhead. This gives enough distance between features that prevent contamination and simplifies the printing alignment process.

### Electrophoresis of synthesized poly(dT)

**Figure 7:**
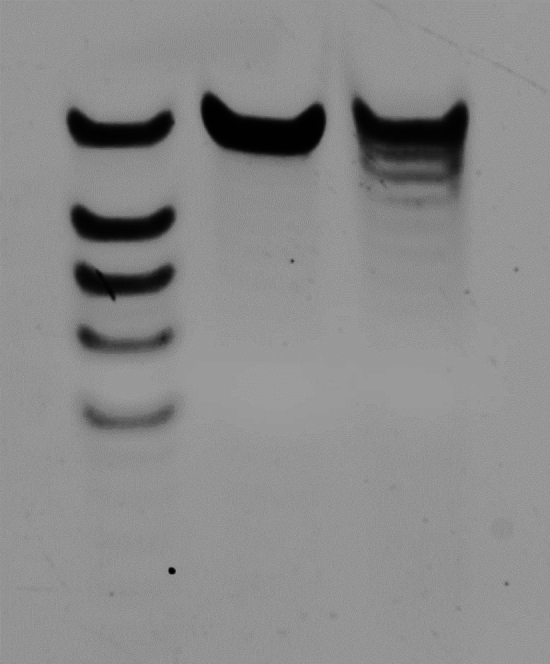
We used 24% urea polyacrylamide gel electrophoresis (urea-PAGE) to analyze the synthesized oligo. 30nt Poly(dT) was synthesized and loaded in the PAGE. Lane 1 is ladders made by mixing poly(dT) 30-, 26-, 24-, 22-, 20nt oligos. Lane 2 is a 30nt poly(dT) as control. Lane 3 is a synthesized 30nt poly(dT) using OpenIDS. It shows the major band is 30nt and some short oligos are existed under the target.

Synthesis of 30nt poly(dT) using OpenIDS was completed in 6 h. Following the post-synthesis process^26^, we obtained some pellets of the oligo, which we diluted in distilled water and analyzed using electrophoresis. Urea polyacrylamide gel electrophoresis (urea-PAGE; 24%) showed the synthetic oligos (Fig. 7). the observed 26~30 nt length band on urea-PAGE, and the binomial distribution formula, the estimated synthesis yield is approximately 98% per cycle. The cost incurred for the 30nt synthesis was approximately $102.61 (Table. S3).

## Discussion

We introduced OpenIDS, an open-source inkjet DNA synthesizer that can be easily constructed even in synthetic biology research labs with limited knowledge of electrical and mechanical engineering. OpenIDS was designed with a focus on simplicity, affordability, and flexibility. To achieve this, most of the structures were 3D printed, and the system was operated by open-source hardware, namely Arduino and Raspberry Pi, which are user-friendly and well-supported by libraries. The synthesizer was sealed with aluminum profiles and acrylic covers, allowing inexpensive cost and easily assembled enclosed synthesizer. The mechanical components are divided into five main modules, which are shown in Figs. 1, 2, 3. The oligo synthesis performance of OpenIDS was tested by synthesizing a 30nt poly(dT) sequence on 144 features. The oligo was then visualized through urea-PAGE (Fig. 7)

As far as we know, OpenIDS is the first open-source inkjet in-situ DNA synthesizer to utilize industrial-grade inkjet printer heads. Other inkjet DNA synthesizers, to our knowledge, have had their structures kept non-disclosed^24^, constructed as manual systems by retrofitting inexpensive office-grade inkjet printers^12^, or formed robust open-source platforms but with the drawback of using office-grade inkjet printheads, many of which are discontinued and no longer supported due to the discontinuation of various components^13^.

Microarray synthesis in individual academic laboratories holds great potential for reducing the cost of microarray production and facilitating challenging research, such as replacing nucleobases with other artificial chemicals. OpenIDS will allow non-experts to fabricate and control a synthesizer quickly and affordably. Moreover, it grants users the freedom to easily add their own printheads or modify the synthesizer board design according to their research purpose. We hope that this flexible modification of the microarray structure and self-synthesis capability will open new horizons in synthetic biology and molecular biology research.

## Methods

### Preparation of reagents

One gram of DMT-dT phosphoramidite (Sigma-Aldrich) was stored at 4 °C, and 1 g ETT activator (Chemgenes) was stored at room temperature in a desiccator. This study used PC as a solvent instead of the conventional solvent, acetonitrile. During the synthesis, humidity had to be totally under control. To ensure the absence of moisture, PC was stored in the desiccator with molecular sieves (3 Å, Sigma-Aldrich) for at least 1 day prior to use. The molecular sieves were stored at 300 °C. We prepared 0.25 M dT and 0.32 M ETT in PC. Before synthesis, the monomer solutions were stored in the desiccator with molecular sieves to maintain their dryness and quality. Detritylation solution (3% trichloroacetic acid in dichloromethane), oxidizer (0.02M I_2_ in THF/pyridine/H_2_O), and ACNbulk solutions were purchased from DUKSAN.

### Silicon wafer substrate preparation

A 4-in silicon wafer with (100) orientation was used in this study. Most of the wafer processing steps were delegated to external agencies. The National NanoFab Center carried out 2000-Å nitride deposition (low-pressure chemical vapor deposition) on wafers. The photomask—produced by Microtec—contains a fine structure of 200-µm square patterns (Fig. 6b). Photolithography was conducted using an AZ GXR 602 photoresist to transfer the fine structure onto the silicon wafer. The photoresist was exposed to the light passing through the photomask and subsequently removed. Then, RIEwas performed at the Korea Institute of Science and Technology Microfab to strip the nitride layer from one side of the wafer. After nitride removal, the exposed silicon was uniformly etched at an angle of 54.74° in the 30% KOH solution to form trapezoidal wells that could hold the CPG in the wafer. Finally, 14 pieces of 15 × 25-mm silicon wafer were obtained from the 4-in wafer. Each piece of wafer contained wells arranged in a 16 × 9 pattern. The well size was optimized to 200 μm, and the distance between each well was 1371 μm, which corresponds to 10 times the center-to-center distance of the print nozzles.

### Synthesizer construction

OpenIDS was built in-house using aluminum profiles and acrylic covers for the framework. To do this, open-source hardware (i.e., Arduino and Raspberry Pi) was integrated into the Python software. Most of the components were 3D printed using a Sindoh 3D printer. Because of the hazardous reagents, every component had to possess chemical resistance. The substrate holder was also 3D printed using PPfilaments. Teflon and silicone tubes were used for the solution flow. A linear actuator moves along the x-axis and delivers the substrate holder to the proper position for each step. A syringe pump (Hamilton, PSD/4) delivers a bulk solution to the substrate on the substrate holder. The actuator and syringe pump are controlled by Arduino. A Raspberry Pi camera observes the progress of the synthesis. Additional sensors, such as pressure and limit sensors, were installed to help ensure that all operations were appropriate during synthesis.

### Inkjet printer-based delivery system

Xaar 128 printheads were purchased from Xaar Corporation (UK). The printhead, which has 128 nozzles, can jet a volume of approximately 40 pL at each nozzle. Integrated software enables simultaneous control of the open-source systems and the Xaar 128 printheads.

It is important to maintain the optimal condition of the inkjet heads to ensure the integrity of the experiments. To prevent nozzle clogging, a 5µm filter was set in front of the head inlet. Additionally, a regular cleaning step was performed to ensure the proper function and longevity of the printhead.

### Poly(dT) synthesis based on the phosphoramidite method

The phosphoramidite method is sensitive to humidity. Thus, before synthesis began, N_2_ or Ar gas was introduced into the OpenIDS to eliminate humidity and air. When charging the dT and activator into the printhead, the reagents should not come into contact with moisture. To prevent any such contact, 3D printed peristaltic pumps were used to deliver the dT and activator reagent into the inkjet head in an enclosed state. Nozzles in the head must be filled with enough solution, with no empty space or bubbles. Every synthesis step proceeded in the enclosed synthesizer, and low humidity was maintained throughout all steps. the reading values of digital hygrometers surge due to the vaporized dichloromethane during synthesis. So, we used an analog hygrometer. we filled synthesizer with excess Argon gas until reaching the minimum reading value. We then synthesized while injecting excess Argon gas to prevent humidity increase during the synthesis process.

Each cycle of synthesis includes (1) coupling, (2) oxidation, (3) coupling, (4) oxidation, (5) detritylation. Between each step, a 10-second wash is included. Coupling, oxidization and detritylation steps were carried out for 2 min each. For improved synthesis yield, coupling in the first cycle was conducted for 5 min. At each step, 3 ml of oxidation solution and detritylation solution were used. For the wash step, 10 ml of ACN was used per step. The capping step was omitted to save time and simplify the synthesis protocol.

After the synthesis was completed, a solution containing the oligos was obtained from the substrate via ammonia cleavage. The ammonia was spread on the substrate and incubated in a 50-ml conical tube at 60 °C for 6 h. The ammonia was then removed through butanol precipitation of the solution, and the oligos were collected and dried in a desiccator. After drying, they could be used for analysis.

### Urea-PAGE of synthesized poly(dT)

We purchased 40% acrylamide-bis solution (19:1), 10×TBE, urea, TEMED, and 10% ammonium persulfate solution (APS) from Biosesang (KR) and prepared 24% urea-PAGE for DNA analysis. TEMED and APS were added simultaneously to a mixture of acrylamide solution, 10×TBE, and urea. After inverting the solution 2~3 times, it was poured into the gel cassette to solidify. Warm 1×TBE solution was used as a buffer to ensure a clear PAGE. We flushed each well to remove the urea for electrophoresis. Gel loading buffer II (Invitrogen) and the samples were mixed 1:1 for a total of 10~20 μl. After electrophoresis, the gel was incubated in a 1×TBE solution with SYBR gold and washed. Urea-PAGE showed every each mers of DNA bands.

### Supplementary Information

Supplementary Information.

## Data Availability

All data generated or analyzed during this study are included in this published article or will be provided by the corresponding authors upon request.
