# Supplementary material for: An open-source, 3D printed inkjet DNA synthesizer
Source: Sci Rep. 2024 Feb 14;14:3773. doi: 10.1038/s41598-024-53944-x (PMC10867077; doi:10.1038/s41598-024-53944-x)
Supplement: Supplementary file 1 — Supplementary Information. [file 41598_2024_53944_MOESM1_ESM.pdf]

Supplementary information

An Open-Source, 3D printed Inkjet DNA Synthesizer

Junhyeong Kim[1,+], **Haeun Kim**[1,+], **Duhee Bang**[1,*]

[1]Department of Chemistry, Yonsei University, Seoul, Korea

[*]Correspondence: duheebang@yonsei.ac.kr

[+]These authors contributed equally to this work.

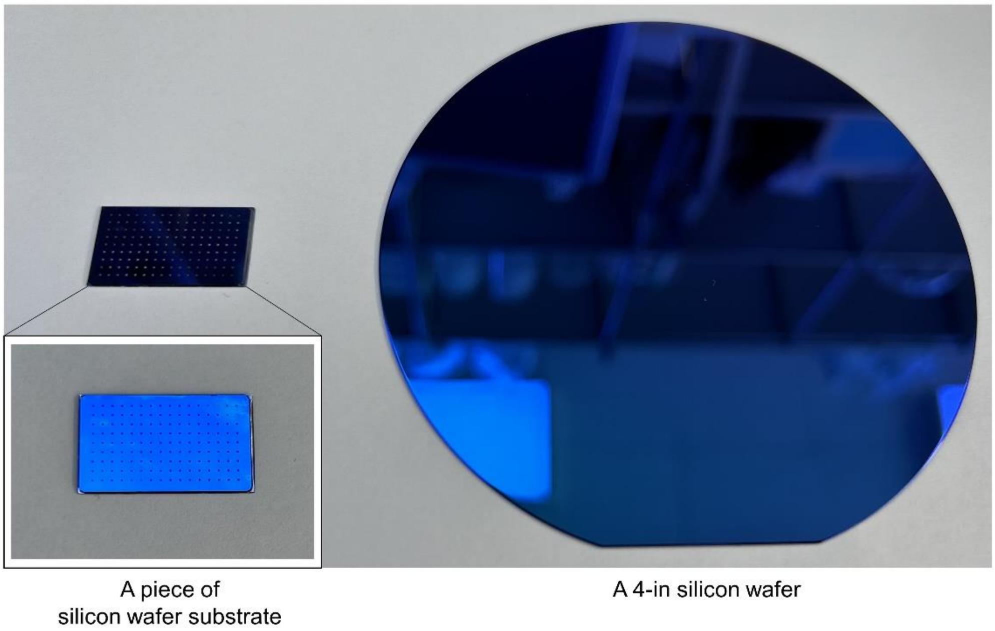

Fig. S1 Image of silicon wafer substrate and silicon wafer. 14 pieces of silicon wafer substrate are derived from a 4-in silicon wafer. Silicon wafer substrate has 9x16 200um features on its surface.

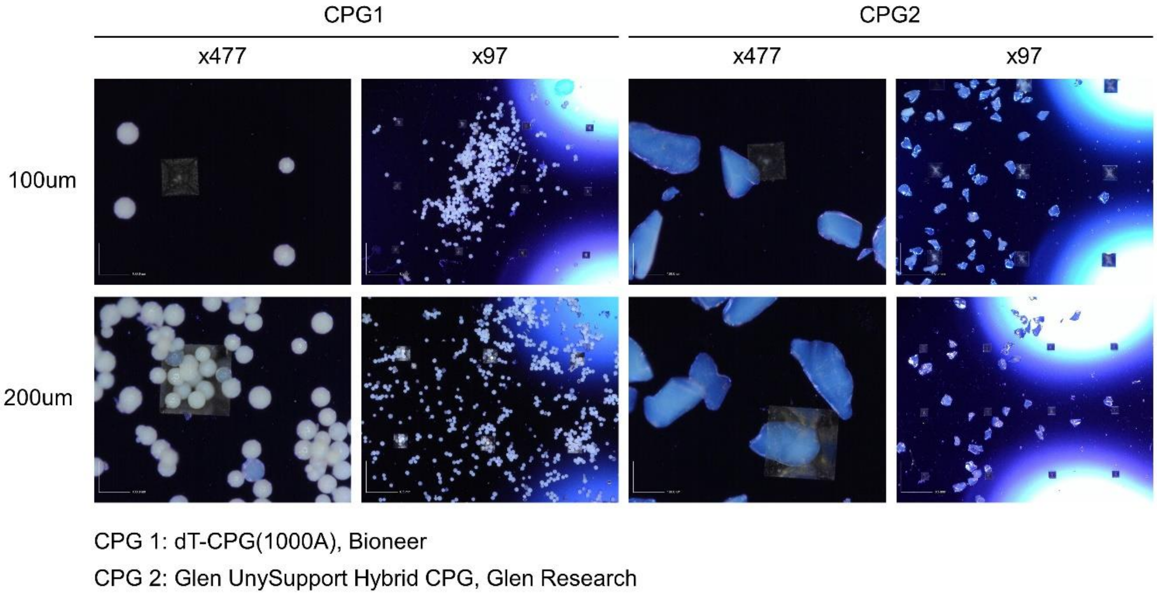

Fig. S2 Silicon wafer compared with CPGs. Squares on the surface are 100- or 200-um features. It was compared with two CPGs. The mean diameter of CPG1 is approximately 70~80um, which is the small size for CPG. 100um feature can contain one or two CPG1. While 200um feature can contain several CPG1. In the case of CPG2, 200um can contain one or two CPG2. However,100um more often not contain CPG2.

Table S1. OpenIDS has several inherent advantages over in situ inkjet DNA Synthesizer

Synthesizer	Open-source	3D-printed	Printhead (grade)	Pitch of feature	Assembly Cost ($)	Synthesis cost for one array ($)
Li et al[1]	X	X	Epson L800 (Office)	1,700um	private	private
Hood et al[2]	X	X	private (industrial)	130um	private	private
POSAM[3]	O	X	Epson F057020 (Office)	280um	34,000	24.68
OpenIDS	O	O	XAAR128 (Industrial)	1,371um	19,891	102.61

1. Li, H. *et al.* An oligonucleotide synthesizer based on a microreactor chip and an inkjet printer. *Scientific reports* 9, 5058 (2019).

2. Hood, L. E. *et al.* High-density oligonucleotide arrays. *Biosensors and bioelectronics* 11.6-7(1996)

3. Lausted, C. *et al.* POSaM: a fast, flexible, open-source, inkjet oligonucleotide synthesizer and microarrayer. *Genome biology* 5, 1-17 (2004).

Table. S2 Components necessary for OpenIDS

Item	Cost Ea. ($)	NO.	Cost ($)
xaar 128/80 L printhead	364.5	5	1822.5
HPC W/CASE XUSB 128	1460	2	2920
XAAR 128 Flexible cable assembly with handle	1458.1	5	7290.5
XUSB Drive electronics system	1216	1	1216
XUSB/XPM PSU 30V 16A	635	1	635
XUSB POWER SUPPLY CABLE 30V	33.4	1	33.4
CABLE USB A-B 2M	10.7	1	10.7
Firewire cable with assembled ferrites	46.8	3	140.4
Mains lead japan 15A black	40.1	1	40.1
aliminium profile	77.59	1	77.59
acrylic cover	671	1	670.9
Foryoutech ballscrew actuator ACT_BLACK_500mm	282.3	1	282.3
Arduino CNC nano shield V4	5	2	10
A4988 Reprap stepper motor driver	2	6	12
Arduino Nano	26	2	52
Arduino Mega 2560	48.4	1	48.4
Raspberry Pi 4 (1GB)	35	1	35
Raspberry Pi HQ Camera Module	73	1	73
300X Microscope lens for Raspberry Pi HQ camera	70	1	70
Autonics PSQ-C01C-RC1/8 air pressure sensor	48.6	5	243
Autonics BS5-Y1M-P photoelectric sensor	7.2	2	14.4
Silicone tubing, 3mm ID x 4mm OD x 2m	6	1	6
Silicone tubing, 3mm ID x 4mm x 10m	100	1	100
3Way Y shaped barbed fitting for 1/8" ID, PP	1	5	5
Nema17 stepper motor, bipolar 1.5A. 42x42x38mm	9	5	45
24V 10A SMPS	18	1	18
12V 10A	13	1	13

TB6600 Step motor driver	10	1	10
HAMILTON™ PSD/6 Precision Syringe Pump Module, Standard, 60mm, 6,000/48,000 steps	1175	3	3525
HAMILTON™ HV 3-2 Valve, "Y" 3-port, PTFE, 0.06"(1.5 mm) port,1/4"-28 Thread, Al/PTFE/CTFE	127	3	381
Super Flangeless™ Ferrule M6 [1/8" OD Tube]	7.6	6	45.6
[Fitting] Super Flangeless™ Nut [1/8" PEEK, M6 Short]	7.6	6	45.6
total			19891.39

Table. S3 Cost of materials required to make 144 x 30nt poly(dT) oligonucleotides,

Item	Purchase	Price ($)	Required	Cost ($)
Propylene carbonate	1,000 ml	17.07	12 ml	0.205
DMT-dT Phosphoramidite	12 X 1 g	66.11	1 g	5.509
5-(Ethylthio)-1H-tetrazole	1g	110.96	0.33 g	36.617
Oxidizer	2,500 ml	48.88	180 ml	3.519
TCA Deblock	2,500 ml	29.56	90 ml	1.064
Acetonitrile 99.5%, EP Grade	4,000 ml	34.14	1,500 ml	12.802
Molecular sieves, 3 Å	1 Kg	145.10	30 g	4.353
Argon	4000 scf	31.04	40,000 scf	31.04
Processed silicon wafer substrate	-	7.5	1 each	7.5
total				102.610

Table. S4 Cost for processing silicon wafer substrate

Step	Cost ($/piece of wafer)	Cost ($/wafer)
silicon wafer	2.1	29.0
LPCVD	1.2	17.5
photolithography	2.6	37.0
RIE	1.6	22.2
total	7.5	105.6

*14 pieces of wafers come from a silicon wafer.

**Constantly usable KOH and photomask is excluded.
